# Sustainable Additive Manufacturing: Mechanical Response of Polyethylene Terephthalate Glycol over Multiple Recycling Processes

**DOI:** 10.3390/ma14051162

**Published:** 2021-03-02

**Authors:** Nectarios Vidakis, Markos Petousis, Lazaros Tzounis, Sotirios A. Grammatikos, Emmanouil Porfyrakis, Athena Maniadi, Nikolaos Mountakis

**Affiliations:** 1 Mechanical Engineering Department, Hellenic Mediterranean University, 71410 Heraklion, Greece; vidakis@hmu.gr (N.V.); mth95@edu.hmu.gr (E.P.); mh90@edu.hmu.gr (N.M.); 2 Department of Materials Science and Engineering, University of Ioannina, 45110 Ioannina, Greece; latzounis@uoi.gr; 3 Department of Manufacturing & Civil Engineering, NTNU-Norwegian University of Science and Technology, Building B’, Teknologivegen 22, 2815 Gjøvik, Norway; 4 Department of Materials Science and Technology, University of Crete, 70013 Heraklion, Greece; maniadi@materials.uoc.gr

**Keywords:** additive manufacturing (AM), three-dimensional (3D) printing, recycling, polyethylene terephthalate glycol (PETG), tensile test, flexural test, Charpy’s impact test, vickers microhardness, scanning electron microscopy (SEM)

## Abstract

The continuous demand for thermoplastic polymers in a great variety of applications, combined with an urgent need to minimize the quantity of waste for a balanced energy-from-waste strategy, has led to increasing scientific interest in developing new recycling processes for plastic products. Glycol-modified polyethylene terephthalate (PETG) is known to have some enhanced properties as compared to polyethylene terephthalate (PET) homopolymer; this has recently attracted the interest from the fused filament fabrication (FFF) three-dimensional (3D) printing community. PET has shown a reduced ability for repeated recycling through traditional processes. Herein, we demonstrate the potential for using recycled PETG in consecutive 3D printing manufacturing processes. Distributed recycling additive manufacturing (DRAM)-oriented equipment was chosen in order to test the mechanical and thermal response of PETG material in continuous recycling processes. Tensile, flexure, impact strength, and Vickers micro-hardness tests were carried out for six (6) cycles of recycling. Finally, Raman spectroscopy as well as thermal and morphological analyses via scanning electron microscopy (SEM) fractography were carried out. In general, the results revealed a minor knockdown effect on the mechanical properties as well as the thermal properties of PETG following the process proposed herein, even after six rounds of recycling.

## 1. Introduction

**Figure 1:**
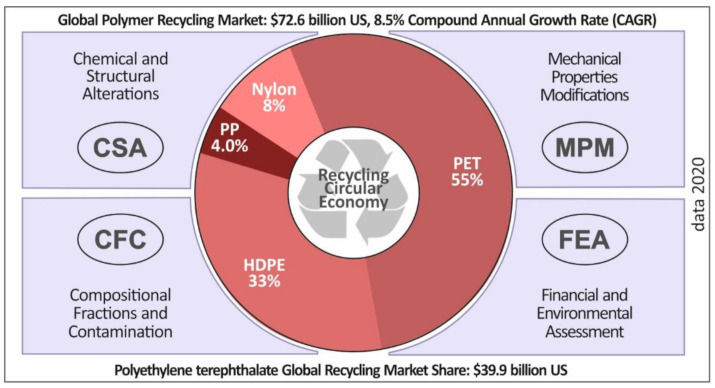
Critical parameters of the global recycling circular economy and the global recycled plastics market volume in 2020 (market volume data source [28]).

Plastic products, including various forms of casings and food/beverage packaging, are manufactured through a wide range of manufacturing processes and are in high demand within the global market [1]. It is therefore of great importance to reduce the carbon footprint of such materials and related manufacturing processes. European Union (EU) Waste Directives impose landfill taxes in order to create appropriate conditions in order to increase the current recycling rates [2]. There is a rather low rate of recycling in the plastics industry, with only approximately 50% of the volume of manufactured plastics reaching recycling processes [3]. Polyethylene terephthalate (PET) is a widely used material, especially in the packaging industry [4]. PET is also a material that has the potential to be used in a wide range of applications, for instance in textiles, plastic foils, membranes, etc. Polyethylene terephthalate (PET) is the most common thermoplastic polymer resin amongst the polyesters mainly used in the food packaging industry. Glycol-modified PET copolyester (PETG) is known to have some enhanced properties over the PET polymer, i.e., chemical alkali resistance, high shrinkage, transparency, gloss, low haze, good printability, etc. Moreover, PETG can be easily vacuumed and pressure-formed, and can be heat-bended thanks to its low forming temperature level(s). Therefore, PETG has recently attracted the interest from the fused filament fabrication (FFF) three-dimensional (3D) printing community as a quite novel and promising material which matches very well with FFF process conditions. In Figure 1, it is shown that PET represents 55% of polymers globally used in recycling processes. This percentage corresponds to a turnover of approximately $42 billion (US) from recycling procedures. A sustainable plan for a serious circular economy implies a balanced energy-from-waste strategy.

The melt blending process is a well-known method in the thermal/melting combined mechanical recycling industry due to its low cost and potential for large-scale processing [5]. An extruder is used to transfer thermal energy through barrel sections to the polymer, driving it to plasticization [6]. The feeding of the process occurs through a rotating screw which leads the polymer through barrel sections, each one with a different temperature. Finally, molten plastic is delivered from the extruder through a nozzle, producing a fixed cross-section extrudate. The thermal stress and viscous shearing applied to the material during extrusion leads to such phenomena as cross-linking, chain branching, and chain scission [7]. Degradation of the polymer is thus partially driven by the physicochemical properties of the extruded material as well as the polymer macromolecular chains’ length and architecture [8]. Other parameters affecting the polymer’s degradation are the extrusion temperatures and the screw’s rotational speed [9]. These parameters are controllable in every industrial extruder used in such processes and should be optimized in advance, resulting in a less destructive effect on the polymer material properties. Namely, processing with high temperatures and rotational speed deteriorates the polymer’s quality, leading to accelerated degradation and finally to a non-processable material due to inhomogeneity in the polymer melt rheological properties, etc. [10].

The prospect of increasing the recycling rate can be found in the Distributed Recycling for Additive Manufacturing (DRAM) theory [11]. Much research has been conducted regarding the potential of producing filaments for additive manufacturing FFF [12,13,14,15,16,17,18]. Three-dimensional printing and a new trend for recycling in distributed manufacturing procedures may help to create a balanced energy-from-waste strategy. In fused filament fabrication (FFF) technology, a polymeric filament of 1.75 mm in diameter (less regularly 2.85 mm) is used to manufacture the requested part [19]. PETG is a modified PET polymer filled with glycol (which allows for better processability in the FFF manufacturing method) which is still able to preserve food compliance [20]. PETG can be produced through recycling processes either from products originally manufactured with PETG or from virgin PET produced by adding glycol [21]. PETG can be used in a wide range of applications due to its superior mechanical properties compared to PET polymer and its ease of use in 3D printing FFF processes. Considering that most food packaging is produced with PET, the potential for increasing the recycling rate (in combination with glycol filling under certain circumstances) exists using DRAM processes.

There is already literature available on the recycling processes and the derived materials properties for other polymers widely used in industrial and engineering applications. Acrylonitrile butadiene styrene (ABS), polypropylene (PP), and high-density polyethylene (HDPE) represent some examples, and the results show the prospects for recycling polymers in order to produce filaments for additive manufacturing [10,22,23]. Thermomechanical stress applied to polymers during filament fabrication seems to have a low effect on mechanical properties, allowing for the repetition of the process over four to five cycles. Similar research conducted with virgin PET under a mechanical recycling procedure [8] showed a rather steep degradation even after the second recycling cycle. The anisotropy of the final parts in the FFF process [24] and the effect of the 3D printing parameters on mechanical properties have been thoroughly studied [25,26], while the effect on recycling on the mechanical and thermal properties of the polymer have not been presented in the literature so far.

In this study, the thermomechanical remelt processing effect of a low-volume recycling process (DRAM) on the mechanical and thermal properties of PETG has been studied. The prospect of reusing PETG in additive manufacturing (AM) applications was studied according to existing recycling models (such as DRAM). The process was chosen to specify the mechanical response of the material even after more than one cycle of recycling. Traditional processes show a great effect on the mechanical and the thermal properties of the polymers, while DRAM-oriented recycling processes, such as that followed in this work, have a totally different effect on the recycled material properties. PETG, which belongs to the PET thermoplastic polymer family as a copolymer derivative, was used in repeated recycling for additive manufacturing filament fabrication.

More specifically, 3D AM printing was chosen, for the purposes of the current study, as a manufacturing method for specimen fabrication in order to study the effect of recycling on the material’s mechanical and thermal properties. It is of great importance to note that the equipment used is DRAM strategy oriented. Although the mechanical response of 3D printed parts seems to be inferior to that of other manufacturing methods, recycling polymers for use in AM applications shows potential for a circular economy. This is of importance considering that 3D printing is an industrial process that is already utilized in industrial applications such as in foundry [27].

In this work, specimens produced using AM were thoroughly tested according to international standards for mechanical, thermal, and morphological behavior in each recycling process. The material was reprocessed six (6) times using same experimental parameters. Tests were conducted for tensile, flexural, and impact-related behavior. Moreover, Vickers micro-hardness was measured in order to make conclusions with regard to the effect of the thermal reprocessing on the material’s mechanical properties. Finally, a thermogravimetric (TGA) test and differential scanning calorimetry (DSC) were performed to determine the effect of recycling on the thermal properties of the polymer, while scanning electron microscopy (SEM) and Raman spectroscopy analyses were conducted in order to study the spectroscopic responses and the specimens’ fractured surface morphological characteristics. The results revealed a rather small effect of thermomechanical stress on the final PETG mechanical properties, even after six rounds of recycling. Thermal and morphological analysis also verified that PETG is an engineering-grade material able to be employed in industrial applications. The DRAM strategy allows for repeated usage of PETG in a variety of applications according to local needs without compromising the mechanical behavior and equipment needed for the recycling process.

## 2. Materials and Methods

### 2.1. Materials

Felfil PETG (Felfil Srl, Torino, Italy) in the form of pellet was used in this work.

### 2.2. Methods

#### 2.2.1. Recycling Process

In this study, a procedure involving a repeated melt extrusion process was followed in order to simulate the degradation mechanism of the recycling process on the polymer. Initially, a filament was produced from the selected material in pellet form. The filament was then used for 3D printing of specimens and to identify the specimens’ mechanical properties according to international standards. The filament then was shredded into fragments. After a drying procedure at 80 °C for 24 h, the pellets were extruded through a single-screw extruder in order to produce the filament for FFF. The produced filament, measuring the desired 1.75 mm in diameter, was then once more dried in the same conditions. Part of it was used to produce 3D printed specimens, while the rest of the filament was shredded back into fragments in order to recycle the material. The above procedural steps were repeated 6 times (6 rounds of recycling).

**Figure 2:**
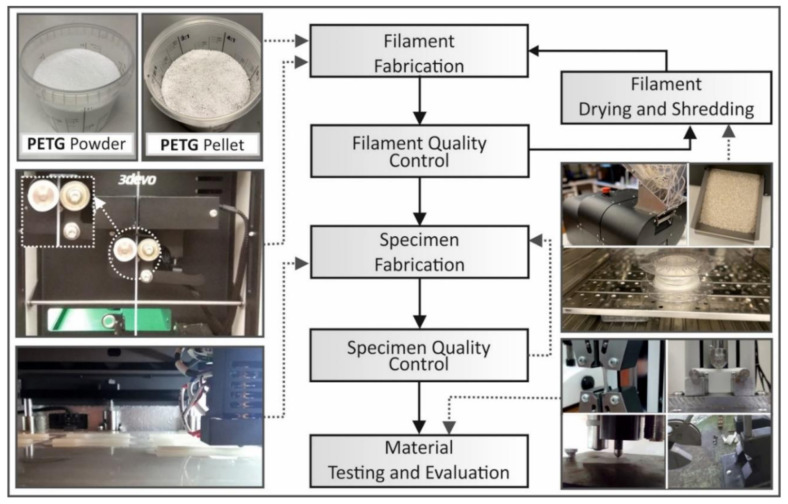
The recycling methodology flow chart followed in this study. PETG: glycol-modified polyethylene terephthalate.

For each round of recycling, filament quality control was checked using a real-time monitoring system working with optical sensors (the extruder’s built-in system). A deviation of under 0.07 mm on average was measured in the produced filament with each round of recycling. Quality control was also conducted using optical and dimensional measures for each manufactured specimen. Figure 2 summarizes all the processes followed for the purposes of this study.

The extruder utilized in this study was a 3D Evo Composer 450 (3D Evo B.V., Utrecht, The Netherlands). The shredder that was utilized in order to reshape the filament into pellets was a Shreddit apparatus (3D Evo B.V., Utrecht, The Netherlands). Through all rounds of recycling the extrusion parameters were kept constant. More specifically, heat zone 1 (closer to extruder’s nozzle) was set to 215 °C, heat zone 2 was set to 215 °C, heat zone 3 was set to 215 °C and finally heat zone 4 (closer to extruder’s hopper) was set to 175 °C. The extruder’s screw rotational speed was set to 4 rpm, while the cooling fans after the nozzle were set to 50%. It has to be mentioned at this point that all of the above extruding parameters were experimentally determined in order to produce a stable diameter, fine quality filament for 3D printing.

**Figure 3:**
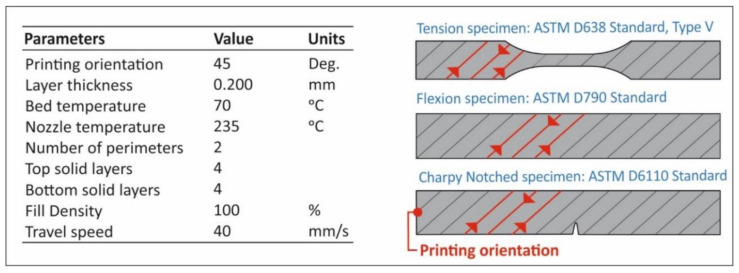
The 3D printing parameters utilized in this study. ASTM: American Society for Testing and Materials.

Fused filament fabrication was selected in order to manufacture specimens for each round of recycling as stated above. Specimens were prepared using an Craftbot Plus (Craftbot Ltd, Budapest, Hungary) FFF technology 3D Printer. The 3D printing parameters are depicted in Figure 3. It should be mentioned that the 3D Printer used in this work was of an all-metal extruder setup and a masking tape (3M 101+, Minneapolis, MN, USA) was used over the aluminum build-plate (heatbed) in order to reduce the wrapping effect on the PETG specimens. Finally, no cooling was used (the nozzle’s fans were set to 0%).

#### 2.2.2. Tensile Specimen Fabrication and Testing

**Figure 4:**
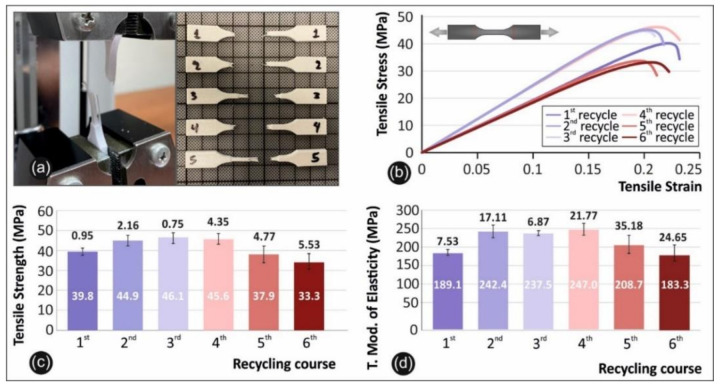
Comparative graphs of the rounds of recycling showing: (**a**) the experimental setup alongside the broken specimens; (**b**) tensile stress vs strain graphs of a specific specimen from each round of recycling (in all cases specimen number 2 was selected); (**c**) the average value and deviation of the tensile strength results for all the studied rounds of recycling; (**d**) and the average value and deviation of the tensile modulus of elasticity values for the studied rounds of recycling.

Tensile testing was carried out under international standard D638-02a of the American Society for Testing and Materials (ASTM) [29]. According to the standard a type-V specimen of 3.2 mm in thickness was chosen, and a total of 6 specimens were printed in 3D and tested. The tensile test equipment used for this purpose was an Imada MX2 (Imada Inc., Northbrook, IL, USA) tension/flexion test apparatus set up in tension mode using standardized grips (Figure 4a). The elongation speed for the tensile test was set up to 10 mm/min as the standard required. Moreover, all the experiments were conducted at room temperature (21 °C).

#### 2.2.3. Flexure Specimen Fabrication and Testing

**Figure 5:**
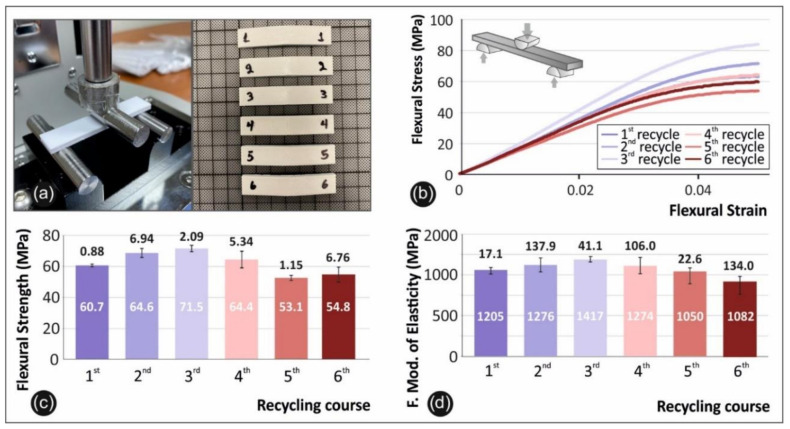
Comparative graphs of the rounds of recycling with: (**a**) the flexure experimental setup alongside the broken specimens; (**b**) the flexural stress vs strain graphs of a specific specimen from each round of recycling (in all cases specimen number 2 was selected); (**c**) the average value and deviation of the flexural strength results for all the studied rounds of recycling; (**d**) and the average value and deviation of the flexural modulus of elasticity values for the studied rounds of recycling.

Three-point bending (3PB) experiments were conducted according to the international standard ASTM D790-10 [30]. A total of 6 specimens were fabricated with a thickness of 3.2 mm and tested using the same equipment as referred to above with regard to the flexion setup. Imada’s chuck speed was set to 10 mm/min and the flexural setup is presented in Figure 5a. Moreover, all the experiments were conducted at room temperature (21 °C).

#### 2.2.4. Impact Specimen Fabrication and Testing

**Figure 6:**
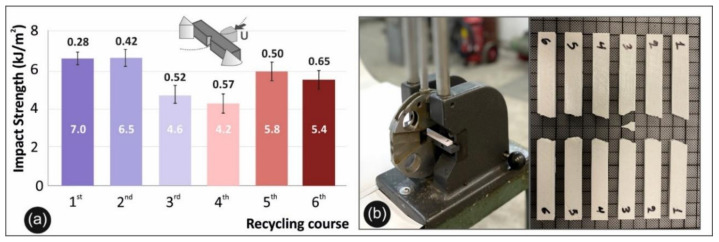
Impact test: (**a**) calculated average values and deviation of the impact strength for all the studied rounds of recycling; (**b**) the experimental setup of Charpy’s impact test with a photo of typical specimens after the tests.

Impact tests were conducted according to the international standard ASTM D6110-04 [31]. Specimens were built with dimensions of 80 mm in length, 8 mm in width, and 10 mm in thickness. A total of 6 specimens were tested in a Charpy impact apparatus. A Terco MT 220 Charpy’s device (Terco AB, Kungens Kurva, Sweden) was chosen for this purpose (Figure 6b). The release height of the apparatus hammer was the same for all the experiments. Moreover, all the experiments were conducted at room temperature (21 °C).

#### 2.2.5. Micro-Hardness Measurements

**Figure 7:**
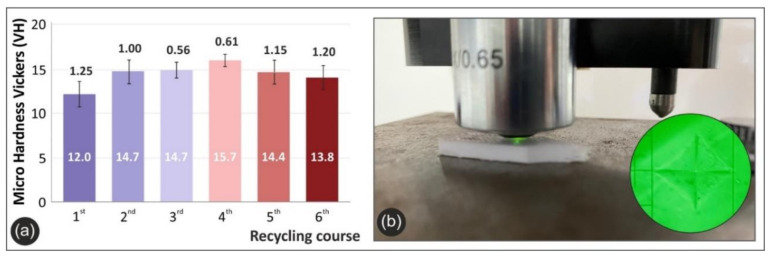
Microhardness measurements: (**a**) The average Vickers micro-hardness values and deviations for all the studied rounds of recycling; (**b**) the experimental setup of Vickers micro-hardness test.

Microhardness measurements were conducted according to the international standard ASTM E384-17. The specimen surface was fully polished before each set of measurements according to the standard’s requirements. An Innova Test 300-Vickers apparatus (Innovatest Europe BV, Maastricht, the Netherlands) was used (Figure 7b). Applied force was set to 100 grF and a duration of 10 s was selected for indentation. Imprints were measured for 6 different specimens for all 6 rounds of recycling. Moreover, all the experiments were conducted at room temperature (21 °C).

#### 2.2.6. Thermal Analysis

Thermogravimetric analysis (TGA) was performed to obtain information about the critical degradation temperature of the virgin PETG material selected for this research in order to identify the appropriate extrusion and 3D printing temperature. The measurements were taken via a Perkin Elmer Diamond Thermogravimetric/Differential Thermal Analysis (TG/DTA) apparatus (Waltham, MA, USA) with a heating cycle of temperature 32 °C to 550 °C, with a heating step of 10 °C/min. Nitrogen was employed as the purge gas.

Differential scanning calorimetry (DSC) was also performed to obtain information about the effect of recycling on the melting point (Tm) and the shift in the degree of crystallinity of the samples. The measurements were taken via a Perkin Elmer Diamond DSC with a temperature cycle of 50 °C to 300 °C, with a heating step of 10 °C/min and then cooling back down to 50 °C. The heating was performed on air.

#### 2.2.7. Raman Spectroscopy

The Raman spectra of the 3D printed PETG samples after consecutive recycling cycles were acquired with the Labram HR-Horiba (Kyoto, Japan) scientific micro-Raman system using a 514.5-nm line of an Ar + ion laser. For all measurements the laser power was set to 1.5 mW at the focal plane to obtain the corresponding Raman spectra. An optical microscope equipped with a 50×-long working distance objective lens was used for delivering the excitation light as well as collecting the back-scattering Raman activity, while all spectra were acquired for the back-scattering geometry.

#### 2.2.8. Morphological Characterization

For the morphological characterization of the internal/external structure and interlayer fusing of the specimens, SEM characterization was conducted. The SEM analysis was performed using a JEOL JSM 6362LV (Jeol Ltd., Tokyo, Japan) electron microscope in high-vacuum mode at 5 kV acceleration voltage on uncoated samples.

Atomic force microscopy (AFM) images were taken on a Park System XE7 apparatus (Park Systems Corp., Suwon, Korea) from the side area of the filament in each round of recycling to evaluate the effect of recycling on the roughness of the material produced.

## 3. Results

### 3.1. Tension Results

**Figure 8:**
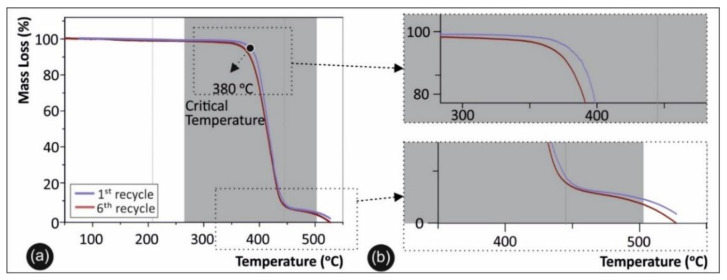
(**a**) Thermogravimetric (TGA) data for pure PETG in the first versus the sixth rounds of recycling; (**b**) magnified area of the TGA degradation curve.

The tensile testing setup utilized in this work is presented in Figure 4 below, along with a photo of typical specimens after the tests (Figure 4a), and comparative graphs of the tensile stress versus strain (Figure 8b), the tensile strength (Figure 4c), and the tensile modulus of elasticity (Figure 4d) for all 6 rounds of recycling.

### 3.2. Flexure Results

In Figure 5 below the flexion testing setup utilized in this work is presented, along with a photo of typical specimens after the tests (Figure 5a) and the comparative graphs of the flexural stress versus strain (Figure 5b), the flexural strength (Figure 5c), and the flexural modulus of elasticity (Figure 5d) for all 6 rounds of recycling.

### 3.3. Impact Results

Charpy’s notched impact test results are shown in Figure 6a. Specifically, in Figure 6a the mean calculated impact strength of each round of recycling for PETG is presented, while in Figure 6b the setup of Charpy’s test is presented.

### 3.4. Micro-Hardness Results

Figure 7a below presents the mean Vickers micro-hardness (HV) values calculated for each round of recycling of PETG, while the experimental setup is depicted in Figure 7b.

### 3.5. Thermal Analysis Results

**Figure 9:**
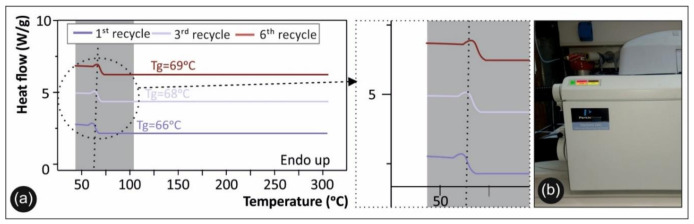
(**a**) Differential scanning calorimetry (DSC) plots of the second heat/cool cycle for PETG after the first, third, and sixth rounds of recycling. (**b**) The device employed for the DSC measurements.

Regarding the thermal analysis, the TGA results for the pure PETG polymer in direct comparison with PETG in the sixth round of recycling are presented in Figure 8a, while a magnification of the degradation area is depicted in Figure 8b. DSC analysis was used for qualitatively defining the degree of crystallinity and the shift in glass transition temperature (Tg) of the studied materials. Figure 9a shows DSC plots of the second heat/cool cycle for PETG after the first, third, and sixth rounds of recycling, along with a magnification of the graph, while the device employed for the measurements is shown in Figure 9b.

### 3.6. Raman Spectroscopic Responses of the Pristine and Recycled PETG Materials

**Figure 10:**
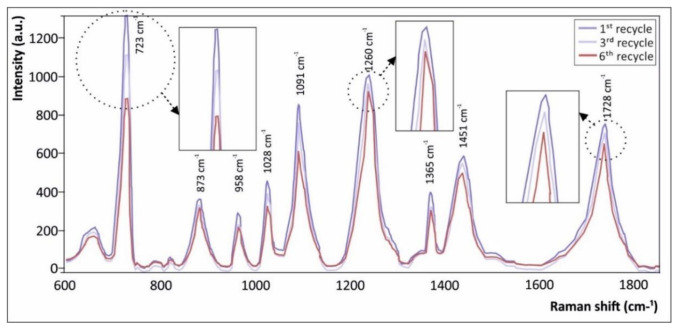
Raman spectra of 3D printed PETG samples utilizing a polymeric filament after the first, third, and sixth rounds of recycling, respectively.

Raman spectroscopy was utilized as non-destructive, rapid, and robust analytical technique to qualitatively demonstrate any possible degradation of PETG from the different consecutive recycling/re-melting processes accompanied by the 3D printing process in each case. Figure 10 illustrates the corresponding Raman spectra of 3D printed PETG samples after the first, second, and sixth recycling cycles. All the acquired spectra presented were treated with a baseline correction through subtraction of a linear or polynomial fit of the baseline from the raw spectra, so as to remove the tilted baseline variation caused by various noises, i.e., fluorescent background, etc.

This is expected to ensure the accuracy of the PETG spectral characteristics, and has been similarly performed in another detailed study where Raman spectroscopy was employed to detect possible degradation of a thermoplastic polypropylene (PP) polymer by consecutive melt-mixing processes [10]. Characteristic common peaks of PETG could be observed at 726 cm^−1^ (C–H ring out-of-plane bending and C=O bending) [32], 873 cm^−1^ (C–H ring out-of-plane bending) [32], 958 cm^−1^ (C–H stretching peak of the cyclohexylene ring found in PETG copolyester) [32], 1028 cm^−1^ (C–H ring in-plane bending) [32], 1091 cm^−1^ (C–O stretching) [33], 1260 cm^−1^ (C–O stretching of ester groups) [34], 1365 cm^−1^ (gauche CH_2_ wagging) [32], 1451 cm^−1^ (–CH_2_ bending peak of the PETG macromolecular chain backbone) [35], and 1728 cm^−1^ (C=O stretching of ester groups) [32].

### 3.7. Microstructural and Roughness Analysis

**Figure 11:**
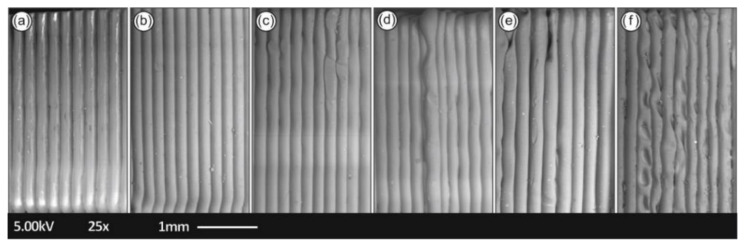
SEM images of the tensile specimens’ side area in the (**a**) first, (**b**) second, (**c**) third, (**d**) fourth, (**e**) fifth, and (**f**) sixth rounds of recycling.

In Figure 11, the SEM images of the tensile specimens’ side areas are depicted in order to identify any faults in the laying of the specimens and to examine the layering interfusion of the specimens in each round of recycling.

**Figure 12:**
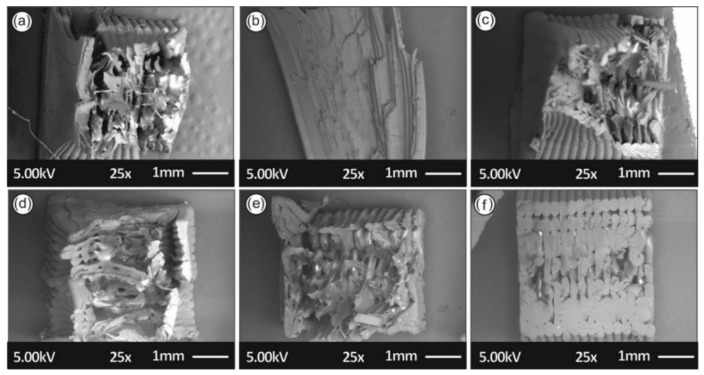
SEM images of the tensile specimens’ fractured surfaces of the (**a**) first, (**b**) second, (**c**) third, (**d**) fourth, (**e**) fifth, and (**f**) sixth rounds of recycling.

In Figure 12, the SEM images depict the fractured surfaces of the tensile specimens, with one for each round of recycling, in order to study the fracture mechanisms and reveal any possible correlation with mechanical property results.

**Figure 13:**
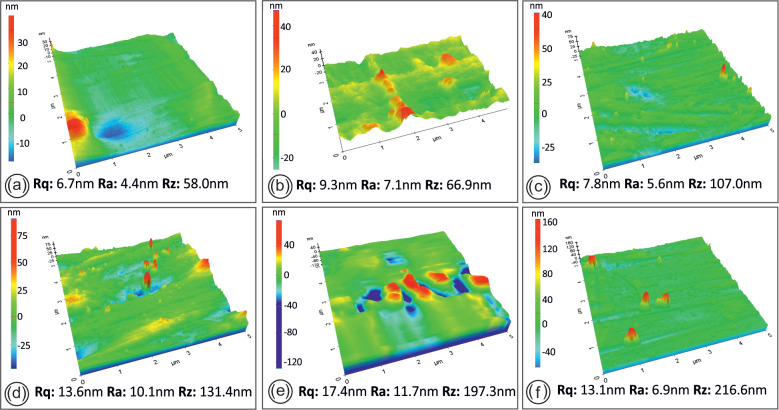
Filament AFM images from each round of recycling; namely after the 1st (**a**), 2nd (**b**), 3rd (**c**), 4th (**d**), 5th (**e**) and 6th (**f**) rounds of recycling.

Regarding the AFM, surface topography and roughness measurements of the side areas of the filament in each round of recycling are shown in Figure 13.

## 4. Discussion

### 4.1. Mechanical Properties

As shown in Figure 4, continuous recycling can cause an overall improvement in the mechanical stability of PETG. More specifically there is a peak value in the third round of recycling, with a value of 46.1 MPa indicating an increase in tensile strength of 15.8% when compared to pure PETG. The tensile modulus of elasticity has a maximum value in the fourth round of recycling, indicating an increase in the elastic modulus of 30.68% when compared to pure PETG. The specimens from the third and fourth rounds of recycling show a stiffer behavior than the other rounds of recycling, revealing a potential strengthening mechanism due to thermomechanical processing during recycling that is eliminated when surpassed by inherent degradation as the rounds of recycling continue. It can be deduced that since PETG is an amorphous polymer, polymeric chains are reoriented or shortened inside the material and this may cause more brittle or ductile behavior.

Reports in the literature show lower or similar values in FFF specimens produced with pure, unrecycled PET, at around 28.12 MPa [36], while literature on recycled FFF PETG reports similar results, with a slight increase in the tensile strength when compared to pure PETG [17]. On the other hand, other studies report a decrease in the tensile strength with rounds of recycling of PETG [32,33,34].

As can be seen from the data of Figure 5, there is a peak value of 71.5 MPa in the third round of recycling, indicating an increase in flexural strength of 17.8%, when compared to pure PETG. The same results are evident for the case of the flexural modulus of elasticity. The peak value is present on the third round of recycling, with an increase of 17.59%, when compared to pure PETG FFF specimens.

Literature reports similar results for the flexural strength of PET and PETG specimens [37,38,39]. To date no research is available on the flexural strength of PETG over continuous rounds of recycling; however, this outcome corroborates the strengthening mechanism revealed from the tensile testing.

Regarding the impact test results, the data depicted in Figure 6 show that in opposition to the tensile and flexural properties there is a clear decrease in the impact strength over the multiple rounds of recycling. This outcome is a potential result of the stiffening of the material, due to the thermomechanical treatment over the consecutive recycling cycles. As such, samples recycled for the third and fourth time reveal a brittle behavior, with higher values when recycling times increase and a potential increase in ductility and internal degradation.

Vickers micro-hardness results confirm all previous findings of material-stiffening and strengthening due to mechanical shredding and multiple melting processes during extrusion and printing. As such, there is an increase of 30.8% in the fourth round of recycling when compared to the material in the first round of recycling.

### 4.2. Thermal Analysis

The TGA results shown in Figure 8 demonstrate the thermal stability and degradation temperature of PETG after the different recycling rounds, revealing any plausible degradation of PETG. Moreover, the TGA plots demonstrate that the maximum operational temperature of the 3D printed samples could be used for engineering thermoplastic materials. Specifically, from the TGA curves information could be extracted about any potential thermal degradation that may have occurred over the different recycling cycles and that could have resulted in a knockdown effect on the material’s thermal stability and mechanical properties. Accordingly, Figure 8b shows a magnified temperature window of the area indicated in Figure 8a; namely, from 340 °C to 500 °C.

As can be observed, all PETG samples exhibited identical values with regard to the “onset of degradation” temperature (Ton), which was found to be ~380 °C. Finally, the TGA curves show that the working temperatures utilized in this study for all PETG samples (for both recycling and 3D printing processes) were below the critical temperature of 380 °C, where in all cases PETG rapidly started to degrade.

Research suggests that there might be a simultaneous occurrence of chain branching or polymer cross-linking along with chain scission when using extrusion systems in the recycling process, while chain scission lowers polymer molecular weights and forms potential byproducts including carbon dioxide, water, and carboxylic acid or aldehyde end groups [40]. Furthermore, changes in the polymer chain formation may result in changes in mechanical properties, i.e., creep, modulus, and hardness, which are presented in continuation. Shorter chain lengths are known to reduce polymer elasticity, embrittle the polymer, and decrease viscosity [20,41,42,43].

### 4.3. Raman Spectroscopic Responses

It can be clearly observed that Raman intensity of the specific bands assigned above gradually decreased with the increased number of recycling cycles. An interesting finding that can be seen in the Raman intensity of the above discussed bands is that for some peaks the decrease was less pronounced from the first to the sixth cycles, specifically the ones at 873 cm^−1^, 1260 cm^−1^, and 1158 cm^−1^, while for some other the decrease was only less pronounced from the third to the sixth recycling cycle i.e., 958 cm^−1^, 1091 cm^−1^, and 1365 cm^−1^. The decrease in intensity may be attributed in general to the possible shortening of the polymeric macromolecular chains that may occur during the multiple extrusion cycles of PETG carried out in ambient conditions. This has been defined as polymer chain scission, which results from various oxidative degradation mechanisms that can occur and that have been previously elaborated [37]. This effect could have a further influence on the molecular weight of the polymeric material. The molecular weight represents the length of the PETG macromolecular chains, which decreases slightly as the degree of degradation increases. This could be the main apparent mechanism explaining a possible knockdown effect of the material’s tensile properties [34]. On the other hand, regarding the peaks where the decrease is less pronounced (from the third to the sixth recycling cycles (958 cm^−1^, 1091 cm^−1^ and 1365 cm^−1^)), a possible explanation is that some melt process-induced crystallization may occur.

The crystallization could then result in the conversion of some of the gauche conformers into trans conformers in the PETG copolyester, corresponding to specific peak absorption as similarly reported elsewhere [32,33]. For example, the conversion of gauche ethylene glycol group conformers to trans conformers causes the bands, as the crystalline trans conformers have a higher relative intensity as compared to those assigned to the amorphous gauche conformers [35].

### 4.4. Morphological Characterization

As can be seen from Figure 11, the interlayering fusion of the FFF specimens changes over each round of recycling. More specifically, the specimens from the fifth and sixth rounds of recycling presented pronounced gaps and deformities between layers in some specimens that could play a significant role in lowering the mechanical properties of the specimens produced. It can be also further deduced that high-quality 3D printed objects were manufactured, with good adhesion between the layers from the first to the fourth rounds of recycling, which is likely to further result in the high mechanical performance of 3D printed components from recycled PETG material. These structural deformities presented in the latter rounds of recycling i.e., the fourth and sixth rounds of recycling, can be attributed to changes in polymer viscosity due to possible crosslinking [41].

As can be seen from Figure 12, the layering interfusion is different for specimens in each round of recycling, as occurs in the failure mode. The best interlayering fusion was found in the specimens from the third and fourth rounds of recycling. For the third and fourth rounds of recycling the specimens seemed to fail in a more ductile manner, with breaking fibers on the filament strands visible in Figure 12c,d. Moreover, filament strands on the specimens from the first, fifth, and sixth rounds of recycling seemed to fail with a much more brittle mechanism, contributing to lower mechanical strength in the cases studied. Regarding the AFM data results in Figure 13, no significant changes in filament roughness (root-mean-square roughness-Rq and roughness average-Ra) were shown with each round of recycling. Filaments from all rounds of recycling were found to contain similar grooves on the side surface that AFM is incapable of accurately measuring. Data results measured from the smoother part of the filaments’ side surfaces showed that there was a slight increase of Rz with each round of recycling.

## 5. Conclusions

**Figure 14:**
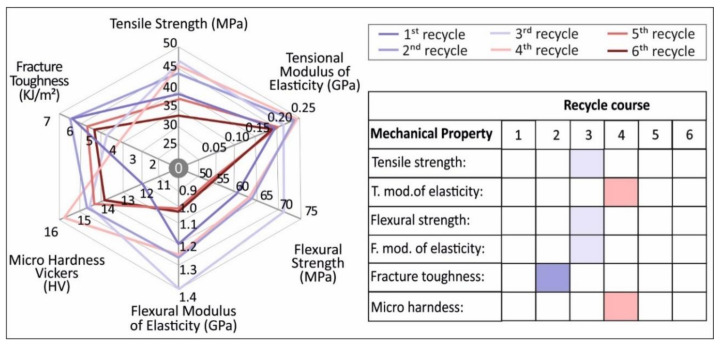
Overall results on the mechanical properties of virgin and recycled PETG in the 6 rounds of recycling studied in this work.

The overall results of this study are depicted in Figure 14. It was shown that the continuous recycling of PETG could slightly improve the overall mechanical strength of PETG FFF specimens, at least up to the fourth round of recycling.

This research was focused on the effect of thermomechanical processing during the recycling process on the mechanical, thermal, and structural properties of virgin PETG over a specific number of rounds of recycling. In total, six rounds of recycling were conducted, and the tensile, flexural, impact-related, and microhardness properties were determined for FFF specimens after each round of recycling. The overall results showed that it was possible to recycle and reuse PETG material multiple times, resulting in a significant gain in the mechanical properties of the recycled materials.

As for the mechanical properties, the fundamental conclusions are as follows:

(1) Thermomechanical processing led to the stiffening and strengthening of the material after the third and fourth rounds of recycling, an effect which did not occur after the fifth and sixth cycles.
(2) There was no significant degradation up until the fifth round of recycling, as revealed by thermal analysis.
(3) The recycled materials were easier to process and print in 3D without issues until the fifth round of recycling, when the polymer flow was significantly decreased.

The results and the outcome of this work will trigger further research endeavors in the direction of optimizing the process for direct industrial use. They also encourage the study of additional polymer materials to evaluate their properties during multiple rounds of recycling for comparison with the PETG polymer studied in this work.

## Data Availability

The data presented in this study are available on request from the corresponding author.
